# Detection of a novel *Pestivirus* strain in Java ticks (*Amblyomma javanense*) and the hosts Malayan pangolin (*Manis javanica*) and Chinese pangolin (*Manis pentadactyla*)

**DOI:** 10.3389/fmicb.2022.988730

**Published:** 2022-09-02

**Authors:** Yuan-Ni Shi, Lin-Miao Li, Jia-Bin Zhou, Yan Hua, Zhi-Liao Zeng, Ye-Pin Yu, Ping Liu, Zi-Guo Yuan, Jin-Ping Chen

**Affiliations:** ^1^College of Veterinary Medicine, South China Agricultural University, Guangzhou, Guangdong, China; ^2^Key Laboratory of Zoonosis Prevention and Control of Guangdong Province, Guangzhou, China; ^3^Guangdong Key Laboratory of Animal Conservation and Resource Utilization, Institute of Zoology, Guangdong Academy of Sciences, Guangzhou, Guangdong, China; ^4^Guangdong Provincial Key Laboratory of Silviculture, Protection and Utilization, Guangdong Academy of Forestry, Guangzhou, Guangdong, China; ^5^Shenzhen Management Bureau of Natural Reserve, Shenzhen, Guangdong, China

**Keywords:** virome, pangolin, *Pestivirus*, *Amblyomma javanense*, molecular epidemiology

## Abstract

Pangolins are endangered animals and are listed in the CITES Appendix I of the Convention International Trade Endangered Species of Wild Fauna and Flora as well as being the national first-level protected wild animal in China. Based on a few reports on pangolins infected with pestiviruses of the Flaviviridae family, *Pestivirus* infections in pangolins have attracted increasing attention. Pangolin pestivirus is a pathogen that may cause diseases such as acute diarrhea and acute hemorrhagic syndrome. To better understand the epidemiology and genomic characterization of pestiviruses carried by pangolins, we detected pestiviruses in dead Malayan pangolin using metavirome sequencing technology and obtained a *Pestivirus* sequence of 12,333 nucleotides (named Guangdong pangolin *Pestivirus*, GDPV). Phylogenetic tree analysis based on the entire coding sequence, *NS3* gene or *RdRp* gene sequences, showed that GDPV was closely related to previously reported pangolin-derived *Pestivirus* and clustered into a separate branch. Molecular epidemiological investigation revealed that 15 *Pestivirus*-positive tissues from two pangolins individuals with a positivity rate of 5.56%, and six *Amblyomma javanense* carried pestiviruses with a positivity rate of 19.35%. Moreover, the *RdRp* gene of the *Pestivirus* carried by *A. javanense* showed a high similarity to that carried by pangolins (93–100%), indicating *A. javanense* is likely to represent the vector of *Pestivirus* transmission. This study expands the diversity of viruses carried by pangolins and provides an important reference value for interrupting the transmission route of the virus and protecting the health of pangolins.

## Introduction

Pangolins belong to the genus *Manis* (family: Manidae, order: Pholidota, class: *Mammalia*). Their bodies are covered with scales and they have no teeth. Pangolins are nocturnal and feed on termites and ants, based on which they are also known as the scaly anteater (Wang et al., 2016). Pangolins are affected the worst among mammals in the global wildlife trade owing to their meat and inflated medicinal properties (Boakye et al., 2015; Gaubert et al., 2018). On the International Union for Conservation of Nature, Red List of Threatened Species, Chinese pangolin and Malayan pangolin are listed as critically endangered; Philippine pangolins (*M. culionensis*), Indian Pangolin (*M. crassicaudata*), Giant Pangolin (*Smutsia gigantea*), and White-bellied Pangolin (*Phataginus tricuspis*) are listed as endangered species; Ground Pangolin (*S. temminckii*) and Black-bellied Pangolin (*P. tetradactyla*) are listed as vulnerable species (du Toit et al., 2017). The habitat of the pangolin is mainly include tropical forests, limestone areas, bamboo forests, broad-leaved forests, and shrubs, in which the ticks live. Ticks are obligate hematophagous ectoparasites of wild and domestic animals as well as humans (Domingos et al., 2013). The Java tick transmits *Anaplasma* spp. (Koh et al., 2016) and novel *Borrelia* spp. (Jiang et al., 2021). There are only few studies available on ticks and tick-borne diseases infecting pangolin species (Khatri-Chhetri et al., 2016). It has been reported that the *Amblyomma javanense* carrying pestivirus may play an important role in spreading diseases of pangolins (Gao et al., 2020).

Viruses have been reported to infect pangolins only in recent years. Pangolins are mainly infected by parainfluenza virus 5 (Wang et al., 2019), canine parvovirus (Chang et al., 2021), pestivirus (He et al., 2022), coltiviruses (Gao et al., 2020), Sendai virus, and coronavirus (Liu et al., 2019). Among them, the detection of pangolin-infecting pestivirus found in the Zhejiang Province, China, indicates that pestiviruses are likely to represent the main pathogen associated with pangolin mortality (Gao et al., 2020; He et al., 2022).

Pangolin pestiviruses, belonging to the genus *Pestivirus* of the Flaviviridae family, are positive-sense single-stranded RNA viruses. They are spherical and enveloped, 40–60 nm in diameter, 11.3–13.0 kb in size, and contain approximately 3,900 amino acids that form a large open reading frame (ORF; Tautz et al., 2015). Pestiviruses mainly infect pigs and ruminants (Vilcek and Nettleton, 2006) and cause severe diseases in animals, such as hemorrhagic diseases in pigs, cattle, and sheep, as well as respiratory diseases (Moenning, 1990). Common pestivirus-associated diseases include classical swine fever (Blome et al., 2017), bovine viral diarrhea (Deregt and Loewen, 1995), and sheep border disease (Righi et al., 2021). These viruses have been detected in wild animals such as bats, bamboo rats, wild boars, bison, giraffes, and wild deer *via* next-generation sequencing (Harasawa et al., 2000; Vilcek and Nettleton, 2006; Wu et al., 2018). Pestiviruses have also been found in arthropods (Harvey et al., 2019; Gao et al., 2020). In general, many wild mammals can carry pestiviruses. Pestiviruses are currently classified into 11 species on ICTV, and pangolin pestiviruses have not yet been clearly classified. Some literature suggests adding eight pestiviruses, among which pangolin pestivirus is classified as pestivirus P (Postel et al., 2021). This also shows that pestivirus research has received more and more attention.

The genome of pangolin pestivirus remains largely unclear, and the mode of transmission is also unknown. Here, we report a new rapidly mutating pestivirus carried by pangolins and their parasitic ticks in Guangdong Province, China. Moreover, we performed molecular epidemiological investigation and variation analysis of pestiviruses carried by pangolins and ticks in Guangdong Province, which may support the protection of pangolins.

## Materials and methods

### Source of pangolins and *Amblyomma javanense*

From August 2019 to May 2021, we collected heart, liver, spleen, lung, kidney, throat swabs, anal swabs, and other tissue samples from pangolins in Guangzhou, Shenzhen, and Heyuan, Guangdong Province. A total of 144 samples were collected from 36 pangolins (11 dead and 25 healthy individuals). A total of 31 parasitic ticks on pangolins were collected from Guangzhou and Shenzhen, Guangdong Province, China from April 2019 to June 2021. All samples were stored in liquid nitrogen and transferred to a laboratory freezer at −80°C. All lab personnel were trained and wore protective clothing for protection against biological agents before sample collection (see Supplementary Table S1 for Sample information).

### RNA extraction

RNAiso Plus (Takara Bio, Dalian, China) was used to extract total RNA from ticks and tissues of pangolins, according to the manufacturer protocols. The tissue samples (50 mg of each sample) were ground into powder in liquid nitrogen and transferred to a DEPC-treated EP tube before being volatilized by liquid nitrogen. The anal and throat swab samples were vortexed for 1 min, and 140 ml of supernatant was collected from each sample after centrifuging at 3,000 *g* at 4°C for 1 min. Viral nucleic acid was extracted using the QIAamp® Viral RNA Mini Kit (Qiagen, Germany) according to the manufacturer’s instructions. Subsequently, 1 μg cDNA was synthesized using the SuperScript III First-Strand Synthesis System (Invitrogen, Waltham, MA, United States) and stored at −20°C in a refrigerator till sequencing.

### Identification of pangolins and ticks

Pangolins were identified based on morphological characteristics. Ticks were identified *via* morphological observation and 16S rRNA sequencing. The primers used were PCTY-F (5′-CTGCTCAATGATTTTTTAAATTGCTGTGG-3′) and PCTY-R (5′-CCGGTCTGAACTCAGATCAAGT-3′; Yang et al., 2021). A 50 μl reaction was set up, containing 25 μl PCR mix (GenTech, Shanghai, China), 22 μl double-distilled H_2_O, 1 μl template, 1 μl forward primer (10 μM), and 1 μl reverse primer (10 μM). T100™ Thermal cycling (Bio-rad, United States) was performed at 94°C for 7 min, followed by 34 cycles at 94°C for 30 s, 45°C for 30 s, 72°C for 1 min, and a final extension of 72°C for 8 min. PCR products were detected on a 1.5% agarose gel and sequenced. In total, 18 sequences of the genus *Amblyomma* were downloaded from the NCBI database (National Institutes of Health, Bethesda, MD, United States). The FTT-NS-2 algorithm was implemented in MAFFT v7.407 to align mitochondrial 16S rDNA of ticks (Katoh and Standley, 2013). Phylogenetic trees based on nucleotide sequences of 16S rDNA were constructed using the maximum likelihood method using the MrBayes approach using the SYM + G nucleotide substitution model (Huelsenbeck and Ronquist, 2001).

### Metavirome sequencing

Reverse-transcribe to double-stranded cDNA using previously extracted lymph node RNA of M1/B1. Synthesis of first and second strands, end repair, and adaptor ligation were performed for library construction, as described previously (Liu et al., 2019). High-throughput sequencing was performed by the Magigene Company (Guangzhou, China). Clean reads were *de novo* assembled using MEGAHIT version 1.0 (Li et al., 2016). BWA (v0.7.17, default parameter: mem-k 30) software was used to align clean reads to the GenBank non-redundant nucleotide (NT) database. Contigs were then classified by BLASTx against the NT database using alignment similarity ≥80%, length of matched area ≥ 500 bp, and *e*-value ≤10^−5^. Contigs with significant BLASTx hits were confirmed as viral sequences (Li and Durbin, 2009).

### PCR verification of whole genome sequence

Multiple alignments were performed with existing pangolin virus sequences in GenBank and specific primers were designed. Reverse transcription-PCR was used to amplify the whole-genome sequence of viruses from M1/B1 Malayan pangolins using lymph node and kidney samples (see Supplementary Table S2 for primer information), and the PCR products were directly sequenced after gel purification and the sequencing results were spliced into the whole genome sequence.

### Genomic characterization

We used CLUSTALW^1^ and Simplot v3.5.1 for genomic similarity analysis of pestiviruses. In total, 25 complete genome sequences of pestiviruses were downloaded from the NCBI database^2^ (See Supplementary Table S3 for sequence information), and phylogenetic tree analysis was performed for polyprotein, nonstructural protein 3 (NS3), and RNA-dependent RNA polymerase (*RdRp*) gene sequences. Viral sequences were aligned using the FTT-NS-2 algorithm implemented in MAFFT v7.407 (Katoh and Standley, 2013). The phylogenetic trees were constructed *via* the maximum likelihood method using the MrBayes approach using the GTR + G or GTR + I + G nucleotide substitution model (Huelsenbeck and Ronquist, 2001). Then, all the ML trees were visualized and exported as vector diagrams with FigTree version 1.4.3.^3^

### Epidemiological investigation

Primers for absolute quantitative PCR detection were designed using the sequence of the Guangdong pangolin pestivirus (GDPV) *E2* gene. The fluorescence quantitative screening primer *E2* gene sequence was ligated into a plasmid to construct a standard template, and a 10-fold dilution was used to construct a standard curve to achieve absolute quantitative screening of pestivirus nucleic acids in the remaining samples. The detection of GDPV in M1/B1 pangolin tissues by absolute quantitative PCR was to find the target organ of the novel virus. Then, the verification of the virus was performed with nested PCR by targeting the *RdRp* gene using the specific primer set. The total volume of the absolute quantitative PCR mixture was 20 μl; this included 10 μl 2× qPCR mixture (Takara Bio), 0.5 μl of forward and reverse primers, 1 μl of template, and the remaining volume included nuclease-free water. The following cycle was used for amplification: 50°C for 2 min; 95°C for 2 min; 40 cycles at 95°C for 15 s, 60°C for 1 min, and 95°C for 15 s; 60°C for 1 min; and 95°C for 15 s (primers are listed in Supplementary Table S2).

## Results

### Identification of pangolin and tick species

**Figure 1:**
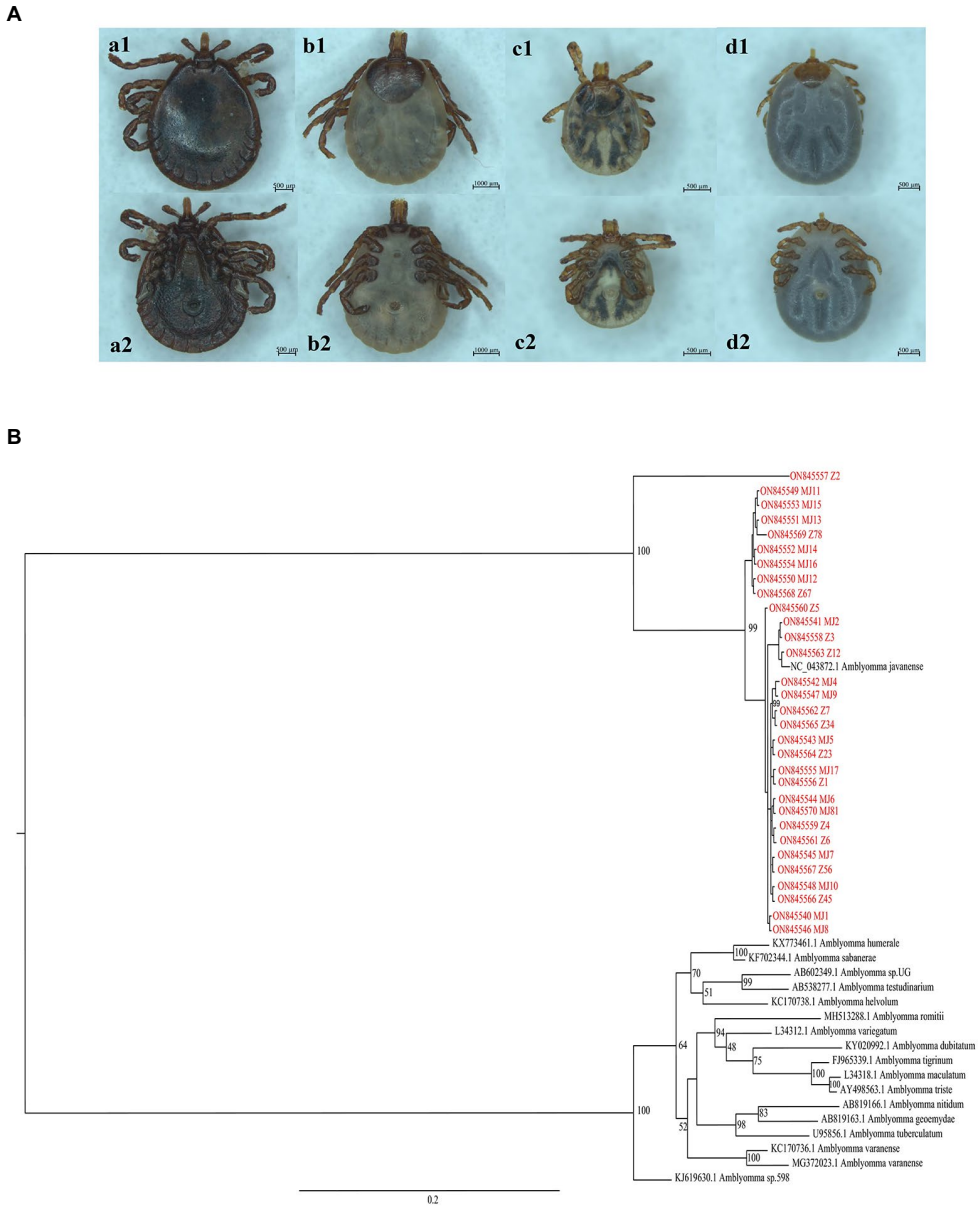
**(A)** View of the dorsal and ventral surfaces of the tick under a stereoscope. a1 and a2 are dorsal and ventral surfaces of male adult ticks, respectively; b1 and b2 are dorsal and ventral surfaces of female adult ticks, respectively; c1, c2, d1, and d2 are dorsal and ventral surfaces of nymphs. **(B)** Phylogenetic trees based on nucleotide sequences of 16S rRNA. The trees were constructed *via* the maximum likelihood method using the MrBayes approach using the SYM + G nucleotide substitution model. Red letters represent the ticks isolated from Guangdong in the present study.

According to body characteristics, there were 20 *Manis javanica* and 16 *M. pentadactyla* species. The ticks were analyzed *via* morphological observation (Figure 1A), PCR amplification and sequencing, and phylogenetic analysis with other tick genera (Figure 1B), and all 31 ticks were identified as *A. javanense.*

### Sequencing results

Sequencing analysis of lymph nodes of M1/B1 pangolin yielded 12.92 Gb of data (28,168,231 clean reads, 150 bp in length). Using BWA software to compare clean reads to virus reference data, a total of 3,708 reads were compared. According to the NCBI taxonomy database, the proportion of DNA viruses compared accounted for 24.93% and the proportion of RNA viruses accounted for 75.07%. Among them, there were six contigs of the genus *Pestivirus* of the Flaviviridae family, with a total of 9,195 nucleotides (nt; Supplementary Table S4; BioProject ID: PRJNA854209).

### Genomic characterization of the novel pangolin *Pestivirus*

**Figure 2:**
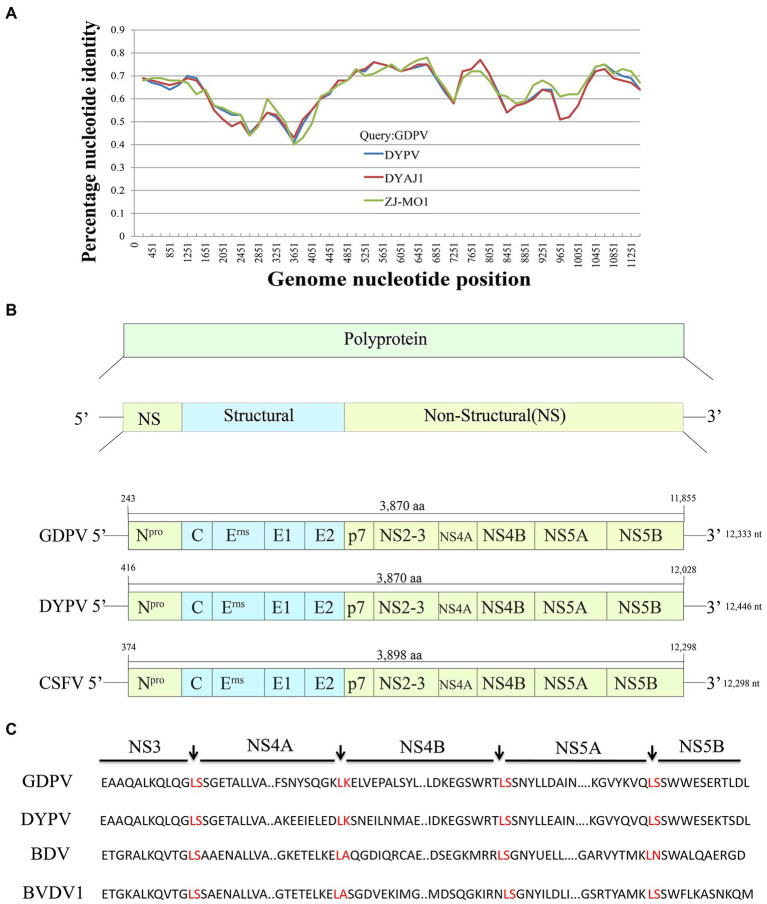
**(A)** Recombination analysis of Guangdong pangolin *Pestivirus* (GDPV) and other pangolin pestivirus based on the whole-genome sequences using Simplot v3.5.10. GDPV, Guangdong pangolin pestivirus isolate M1/B1; DYPV, Dongyang pangolin virus isolate DYCS; DYAJ1, Dongyang pangolin virus isolate DYAJ1; ZJ-MO1, pangolin *Pestivirus* 3 isolate ZJ-MO1. The analysis was performed with the Kimura model, with a window size of 11, 613 bp and a step size of 150 bp. **(B)** Comparison of GDPV genome structure with that of other *Pestivirus* genomes. GDPV, Guangdong pangolin pestivirus isolate M1/B1; DYPV, Dongyang pangolin pestivirus isolate DYCS; CSFV, Classical swine fever virus. **(C)** All cleavage sites of NS3 protease in GDPV. GDPV, Guangdong pangolin pestivirus isolate M1/B1; DYPV, Dongyang pangolin virus isolate DYCS; BDV, Border disease virus; and BVDV1, Bovine viral diarrhea virus 1.

By performing PCR amplification, we obtained the whole genome sequence of GDPV isolate M1/B1 lymph and kidney tissue, which contains a total of 12,333 nt (The GenBank number: ON843279). The genomic similarity analysis with other known pestiviruses showed that the genome sequence of the virus had >40% nucleotide similarity and > 45% amino acid similarity with other known pestiviruses (Table 1). Low similarity and no recombination with other pangolin pestiviruses were found (Figure 2A). GDPV, like other pestiviruses, contains a single long ORF and encodes 12 proteins simultaneously (Figure 2B). Moreover, it encodes two proteins unique to pestiviruses, namely Npro and Erns. Among them, NS3 serine proteinase serves as a molecular model of the polyprotein cleavage site, cofactor requirements, and enzymes necessary for viral replication. Based on the characteristics of the existing *Pestivirus* NS3 cleavage sites (a conserved leucine at the P1 position of all four cleavage sites), followed by serine or alanine (Xu et al., 1997), all cleavage sites were found in NS3 of GDPV (Figure 2C) located at L2474-K2475. Among them, the NS4A/NS4B cleavage site of Dongyang pangolin pestivirus is located at L2315/K2316; the NS4A/NS4B cleavage site of bovine viral diarrhea is located at L2426/A2427.

**Table 1:** Genome-wide homology analysis of GDPV and other pestiviruses.

No.	**Virus** ^*^	% identity with virus no.^#^
		1	2	3	4	5	6	7	8	9	10	11	12	13	14
1	GDPV		67.5	67.6	68.1	41.7	42.0	40.5	42.9	43.0	42.8	40.9	42.5	39.6	42.5
2	DYPV-DYCS	77.6		98.2	70.6	41.1	43.5	39.5	42.1	41.8	41.1	40.0	43.0	38.7	42.2
3	DYPV-DYAJ1	78.0	98.7		71.0	41.3	43.9	39.6	42.1	42.2	41.3	40.2	43.2	39.0	42.4
4	Pangolin pestivirus 3	77.6	80.5	81.0		40.9	41.6	37.7	42.2	40.2	41.0	40.0	43.0	38.3	41.5
5	CSFV	46.8	46.7	46.8	47.1		42.6	58.3	61.3	62.3	67.5	51.1	43.6	62.5	67.9
6	LV	47.2	47.1	47.2	46.4	48.7		45.2	44.3	43.3	43.0	42.1	59.8	38.8	42.9
7	BVDV1	45.1	38.4	45.9	38.0	70.7	49.7		64.6	62.4	59.9	50.1	44.0	58.2	60.1
8	BVDV2	46.1	46.3	46.5	46.1	70.5	49.0	75.5		62.1	61.3	51.3	43.2	59.9	61.3
9	BVDV3	46.5	46.8	47.0	46.3	72.2	48.9	71.9	71.2		62.0	50.8	43.2	62.1	62.6
10	BDV	46.3	46.1	46.3	46.4	78.4	48.5	71.4	71.4	72.1		50.7	43.7	63.3	67.5
11	PAPeV	47.0	40.1	40.1	47.4	57.4	49.0	56.8	57.3	57.7	57.3		42.1	47.8	50.7
12	PPeV	47.8	47.5	47.8	47.8	49.1	68.5	50.4	50.2	49.2	49.5	44.3		39.8	43.5
13	GPeV	46.4	45.9	46.0	46.2	72.2	47.8	68.9	71.3	72.0	72.7	57.0	47.8		63.0
14	AydinPev	47.2	47.0	47.0	47.3	80.1	48.7	71.9	71.3	73.1	79.2	57.3	50.0	73.2	

# Virus Abbrev. Consistent with the International Committee on Taxonomy of Viruses classification.

* Nucleotide similarity is indicated above the diagonal and amino acids similarity is indicated below the diagonal.

### Phylogenetic analysis of a novel pangolin *Pestivirus*

**Figure 3:**
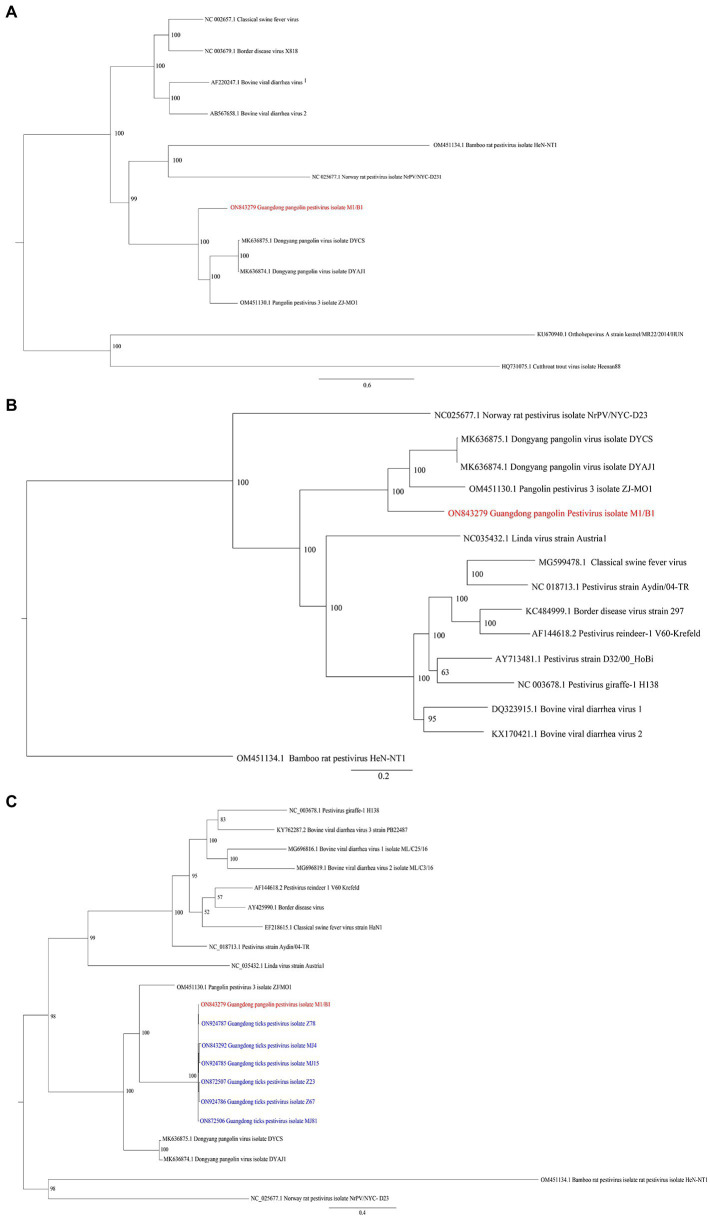
Phylogenetic trees based on nucleotide sequences of **(A)** polyprotein, **(B)** *NS3* gene, and **(C)** *RdRp* gene. The trees were constructed *via* the maximum likelihood method using the MrBayes approach using the GTR + G **(A,B)** or GTR + I + G **(C)** nucleotide substitution model. Red letters represent pestiviruses isolated from M1/B1 pangolins in this study. Blue letters represent pestiviruses isolated from *Amblyomma javanense* from Guangdong in the present study.

The whole-genome sequences of different pestiviruses were downloaded from GenBank to explore the evolutionary relationship between GDPV and other pestiviruses in this study. A phylogenetic tree was constructed based on the nucleotide sequences of polyprotein, *NS3*, and *RdRp* genes. Phylogenetic analysis based on polyprotein (Figure 3A) and *NS3* gene (Figure 3B) showed that GDPV clustered with three other pangolin *Pestivirus* strains. Based on the evolutionary tree of *RdRp* genes, certain *RdRp* genes of Guangdong tick *Pestivirus* (named Guangdong ticks *Pestivirus*, GDTPV) were clustered with that of GDPV (Figure 3C). Further, certain *RdRp* nucleotide sequences of GDTPV showed 93–100% similarity with that of GDPV (Supplementary Table S5).

### Virus screening results

**Figure 4:**
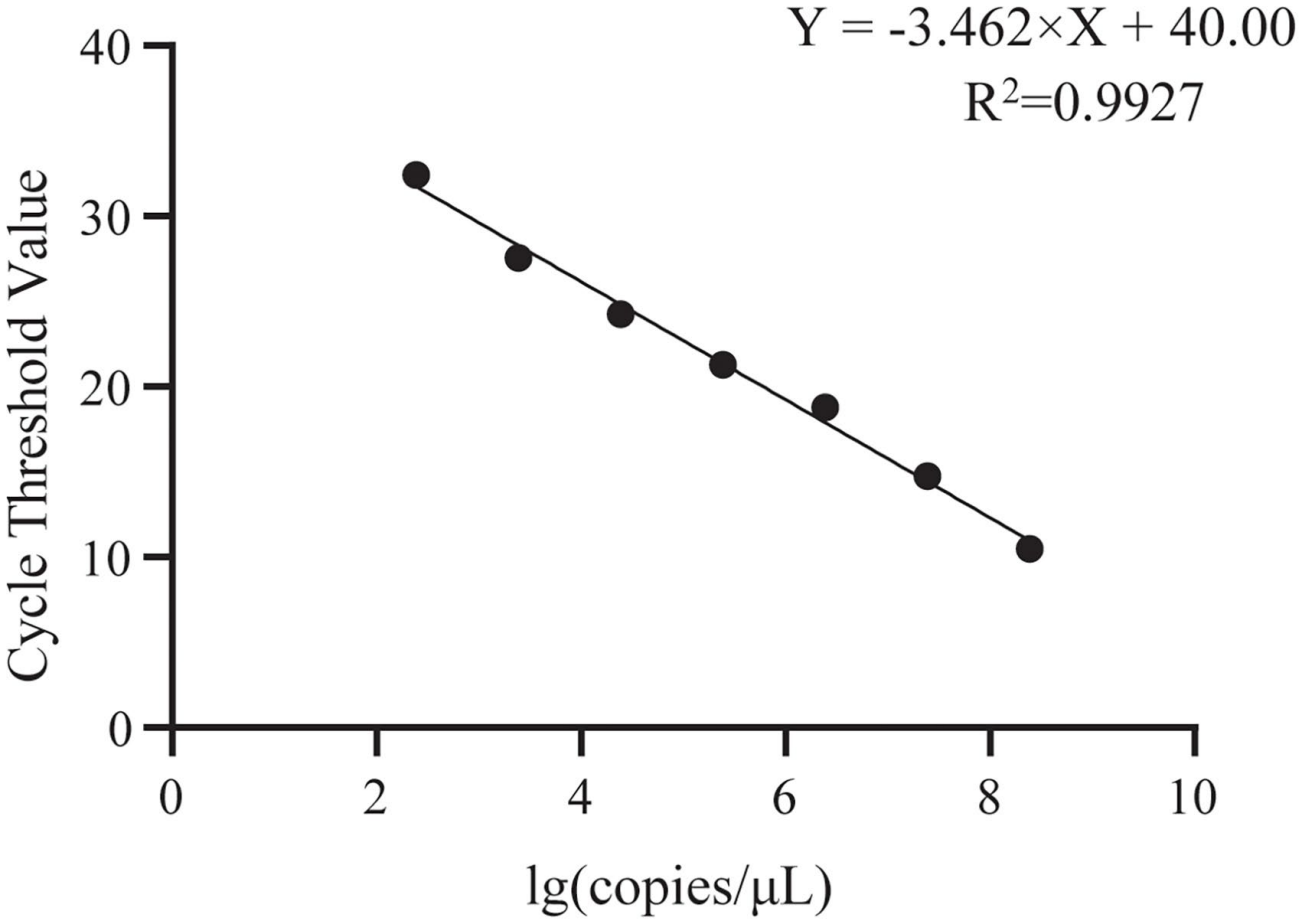
Standard curve for GDPV detection using single-plex qRT-PCR. Copies number/μl of GDPV from 1 × 10^8^ to 1 × 10^2^ copies/μl.

Absolute quantitative PCR designed based on the *E2* gene was performed to detect the presence of GDPV in pangolin samples. According to the results, it was found that the mentioned first melting temperature (Tm) of GDPV was 78°C ± 0.5°C, the slope = −3.462, and the *R*^2^ = 0.9927, which had a good linear relationship. The amplification efficiency of GDPV was 95% according to the calculation formula of amplification efficiency (Supplementary Figure S1; Figure 4). GDPV was detected in M1/B1 pangolin-derived heart, liver, spleen, lung, kidney, pancreas, stomach, and lymph nodes. The *Pestivirus* nucleic acid content in the kidney was approximately 500-fold higher than that in the heart and liver. This indicated that the kidneys are likely to represent target organs of GDPV (Table 2).

**Table 2:** Copies number/μl of GDPV.

**Tissues**	**Cycle threshold value**	**Copies number/μl of GDPV**
Heart	33.06	100.87
Liver	32.47	150.14
Spleen	28.43	2202.24
Lung	26.37	8661.80
Kidney	23.72	50540.50
Pancreas	28.02	2896.49
Stomach	26.44	8234.85
Lymph	31.45	294.90

Nested PCR screening of the *RdRp* gene revealed another *Pestivirus* in D6 pangolins, named Guangdong pangolin *Pestivirus* 2 (Guangdong pangolin pestivirus 2 isolate D6, GDPV2), which was found in liver, spleen, lung, and kidney, duodenum, rectum, and anus: a total of seven tissues. Pestiviruses were detected in six ticks labeled as Mj4, Mj15, Z23, Z67, Z78, and Mj81. We also tested a total of 25 healthy pangolin samples and found no pestivirus. To sum up, we analyzed a total of 15 tissues (M1/B1 and D6 pangolins), with a positivity rate of 10.42%; six ticks carried *Pestivirus* with a positivity rate of 19.35%.

## Discussion

Since the emergence of the new coronavirus in 2019, the possibility of disease outbreaks caused by the consumption of wild animals should be considered (Turcios-Casco and Cazzolla Gatti, 2020). Research on zoonotic pathogens represents a hot topic. As the most smuggled mammal, pangolins are prone to contact with other wild animals and are susceptible to pathogens that cause disease and death. As another blood-sucking parasite, the mosquito may carry several viruses such as Japanese encephalitis virus (JEV) and Getah virus (GETV). And the mosquito-borne viruses have been reported in another pangolin virome study recently (Zhang et al., 2022). The trafficking of pangolins has caused almost irreversible damage to their population (Hu et al., 2020). Chinese customs have made great contributions to intercepting a large number of smuggled pangolins. Due to the lack of research on pangolin diseases and the lack of knowledge about their stress management, rescued pangolins often suffer from poor health and even death. Research on pathogens that infect pangolins will assist in the prevention and treatment of pangolin diseases. The characteristics of many new viruses have been revealed *via* viromics analysis, especially in the monitoring of viruses that infect wild animals. An in-depth understanding of the viruses that infect wild animals plays an important role in the prediction of viral zoonotic outbreaks. For example, viromics studies on bats have revealed more than 130 viruses that infect bats (Hu et al., 2017) including several newly discovered pathogens threatening the public health, e.g., the Ebola virus (Leroy et al., 2005), severe acute respiratory syndrome virus (Wang et al., 2006), and Marburg virus (Swanepoel et al., 2007). Similarly, by performing viromics analysis of pangolins, we identified a potential pathogen.

*Pestivirus* are newly discovered viruses in pangolins. Pestiviruses have been found to be detected in dead pangolins alone and not in healthy individuals (He et al., 2022). A total of 11 dead pangolins and 25 healthy pangolins were investigated for pestivirus-carrying status, and only two dead pangolins were found to be carrying pestivirus. However, none of the healthy pangolins were tested positive for pestivirus. Therefore, *Pestivirus* infections are likely to represent one of the causes of pangolin deaths, which were supported by our epidemiological survey. The phylogenetic trees of the GDPV polyprotein, *NS3*, and *RdRp* genes constructed in this study showed that the Guangdong pangolin *Pestivirus* formed a separate branch and had a low similarity with other *Pestivirus* gene sequences. The nucleotide similarity of GDPV and other pangolin pestiviruses was low (66–69%), which proves that GDPV is a new strain of pangolin pestivirus. Notably, GDPV2 detected in the D6 pangolin was detected using the nested PCR method designed based on the *RdRp* gene, but not by the quantitative PCR assay designed based on the *E2* gene, indicating that the *E2* gene sequence differed between GDPV2 and GDPV. The same was true for pestiviruses detected in ticks that were not detected *via* real-time PCR, but were detected *via* nested PCR. This further shows that the specificity of the *E2* gene was relatively higher and the *RdRp* gene is relatively conservative. However, since the whole-genome sequence of GDPV2 was not verified, its similarity could not be determined, although GDPV2 may represent a new strain of pangolin *Pestivirus*. We also detected the canine parvovirus in the spleen tissues of D6 pangolins (data not shown), further indicating that the viruses carried by pangolins are diverse. Additionally, GDPV nucleic acid content was determined in various tissues of pangolins and we found that the kidneys and pancreas contained the highest GDPV content, which provided some insights into the tropism of *Pestivirus* into pangolins.

The distribution of *A. javanense* in China is mainly concentrated in Fujian, Guangdong, Hainan, and Yunnan. The main host of *A. javanense* is pangolins (Duan et al., 2020), and the pathogens carried by these ticks need to be studied to control the spread of pangolin diseases. It has been reported that *Pestivirus* isolated from ticks that parasitize pangolins only show a 2% nucleotide difference compared with pangolin-derived pestiviruses (Gao et al., 2020). In this study, six *Pestivirus-*positive *A. javanense* were found by screening for *RdRp* gene sequences, and the sequencing results showed that the *Pestivirus* in *A. javanense* was also very close to GDPV. This strongly supports the possibility that *A. javanense* plays a possible role in the transmission of viruses in pangolins and is likely to represent the transmission vector of viruses. In addition to parasitizing pangolins, the *A. javanense* parasitizes wild boars and reptiles such as pythons, monitor lizards, and turtles (Jabin et al., 2019). Tick-related diseases in wild animals should be monitored. Due to low similarities between GDPV2 and GDPV, the whole-genome sequence verification and assembly of GDPV was a great challenge, which is also limited by the sources of sample. Besides, the limitation of pangolin and tick sample sources also affected the epidemiological analysis.

## Conclusion

Pangolins carry various viruses; viruses are killing pangolins. Multiple pangolin pestiviruses were found with low similarities as they mutate rapidly. Further investigation on the virus status of wild animals and their parasites, like pangolins and ticks, is urgently needed. And the better understanding of the spreading ways and the underlying pathogenesis of the viruses carried by wildlife is absolutely important for the public health safety. The present study expands the knowledge on the viral spectrum of pangolins and has an important role in the conservation and public health of rare wildlife.

## Data availability statement

The datasets presented in this study are deposited in the gen bank repository, accession number PRJNA854209, https://www.ncbi.nlm.nih.gov/bioproject/?term=PRJNA854209.

## Ethics statement

The animal study was reviewed and approved by the Committee on the Ethics of Animal Experiments of the Institute of Zoology of Guangdong Academy of Sciences.

## Author contributions

J-PC and Z-GY contributed to the conception of the study. J-BZ, Z-LZ, and YH collected the samples. Y-NS, L-ML, and J-BZ performed virus detection and sequencing. Y-NS and L-ML performed the metagenomics analysis. Y-NS, PL, and L-ML analyzed the data. Y-NS wrote the manuscript. L-ML, Y-PY, Z-GY, and J-PC revised the manuscript. All authors contributed to the article and approved the submitted version.

## Funding

This study was supported by the GDAS Special Project of Science and Technology Development (2020GDASYL-20200103090), the National Natural Science Foundation of China (31972707), and the 2022 Provincial financial special project for Ecological Forestry construction.

## Conflict of interest

The authors declare that the research was conducted in the absence of any commercial or financial relationships that could be construed as a potential conflict of interest.

## Publisher’s note

All claims expressed in this article are solely those of the authors and do not necessarily represent those of their affiliated organizations, or those of the publisher, the editors and the reviewers. Any product that may be evaluated in this article, or claim that may be made by its manufacturer, is not guaranteed or endorsed by the publisher.

## Footnotes

^1^ https://www.genome.jp/tools-bin/clustalw

^2^ https://www.ncbi.nlm.nih.gov/

^3^ http://tree.bio.ed.ac.uk/software/figtree/

## Supplementary material

SUPPLEMENTARY FIGURE S1 **(A)** Gradient amplification curve for GDPV detection using single-plex qRT-PCR, from 1 × 108 — 1 × 102 368 copies/μl; **(B)** Dissolution curve for GDPV detection 369 using single-plex qRT-PCR, Tm value is 78 ± 0.5°C.
