# Supplementary figures and images for: Detection of a novel Pestivirus strain in Java ticks (Amblyomma javanense) and the hosts Malayan pangolin (Manis javanica) and Chinese pangolin (Manis pentadactyla)
Source: Front Microbiol. 2022 Sep 2;13:988730. doi: 10.3389/fmicb.2022.988730 (PMC9479695; doi:10.3389/fmicb.2022.988730)

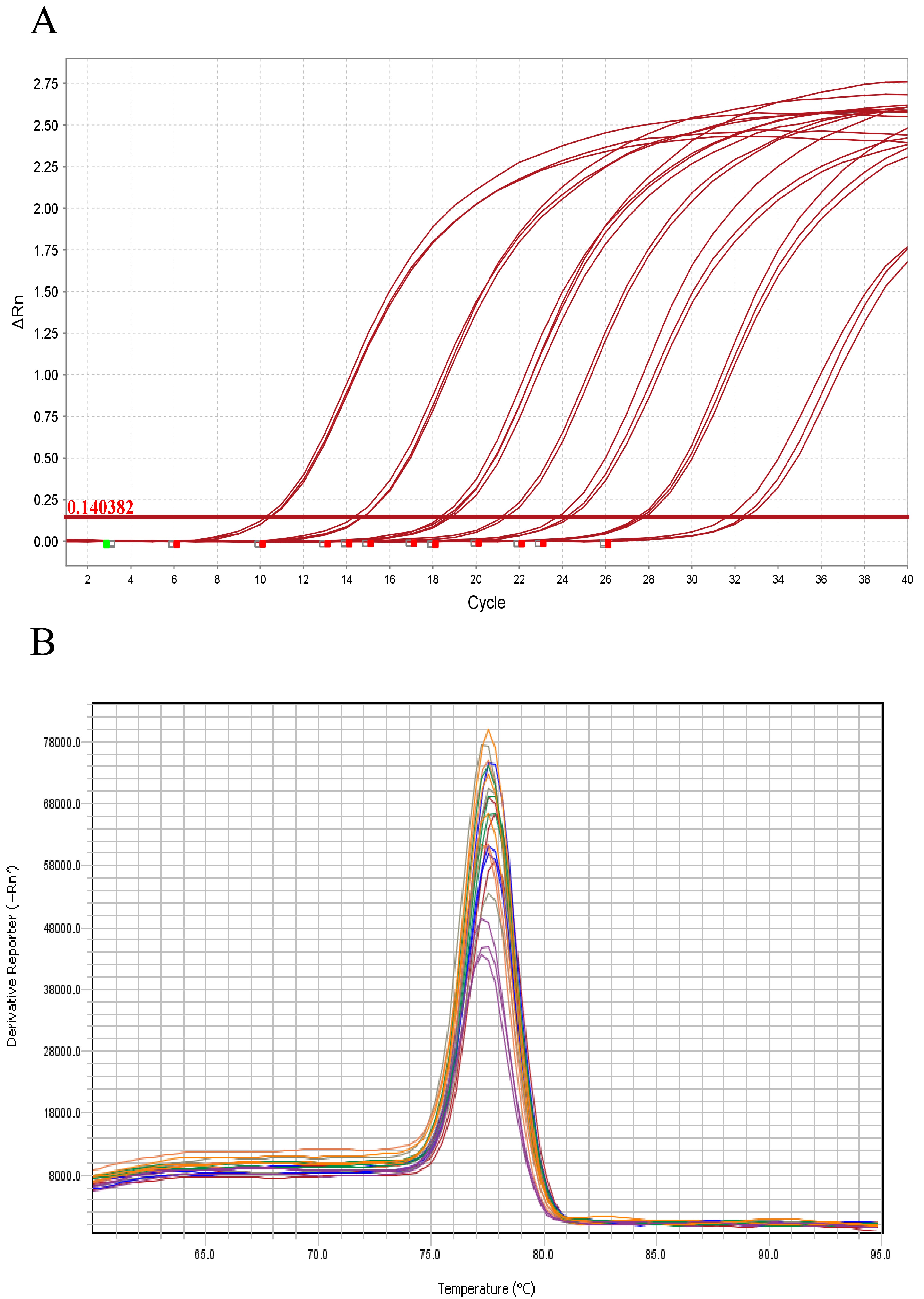

Supplement: SUPPLEMENTARY FIGURE S1 — (A) Gradient amplification curve for GDPV detection using single-plex qRT-PCR, from 1 × 10^8^ — 1 × 10^2^ 368 copies/μl; (B) Dissolution curve for GDPV detection 369 using single-plex qRT-PCR, Tm value is 78 ± 0.5°C. [file Image_1.JPEG]
